# Lactic Acid in Dyspepsia

**Published:** 1855-01

**Authors:** 


Lactic Acid in Dyspepsia.—Dr. C. Handfield Jones, in the Association Medical Journal, advises the use of lactic acid in dyspepsia. He has chiefly given it in cases of irritative dyspepsia, where the digestion was painful and imperfect, and had been so for some time; he does not advise its use at the commencement of the treatment in a severe case, but only after the irritation and vascular erethysm is somewhat reduced. It should be employed in doses of fifteen to twenty minims, in a half ounce of water, and taken at meal times; he states that it seems to mingle with the food, and to supply one of the constituents of healthy gastric juice, which is probably imperfectly produced. Its use need not be confined to cases of dyspepsia, but may be extended to all cases where it is desirable to improve the tone and power of the stomach. It is pleasant, occupies but little space, and the only objection to its use is its present high price; but if much employed it could probably be obtained cheaply.—*Dublin Hospital Gazette.*

